# Sustainable Management of *Tetranychus urticae* and *Trialeurodes vaporariorum* on Tomato and Cucumber Plants Using Rhamnolipids and Essential Oil-Based Biocontrol Agents

**DOI:** 10.3390/insects15090720

**Published:** 2024-09-20

**Authors:** Thomas Thomidis, Petros Damos

**Affiliations:** 1 Department of Human Nutrition and Dietetics, International Hellenic University, 57001 Thessaloniki, Greece; 2 Directorate of Secondary Education of Veroia, Ministry of Education, Religious Affairs and Sports, 59132 Ergohori, Greece; petrosdamos@gmail.com

**Keywords:** biosurfactants, biopesticides, essential oils, Petir Kilat, integrated pest control

## Abstract

**Simple Summary:**

Rhamnolipids (RLs), biosurfactants produced by *Pseudomonas aeruginosa*, were evaluated for their efficacy against two key pests, the two-spotted spider mite and the greenhouse whitefly, in comparison to commercial biopesticides (abamectin and NATURALIS) and a novel essential oil-based product (Petir Kilat). Conducted in a greenhouse setting on cucumber and tomato plants, the study found abamectin to be the most effective, rapidly reducing spider mite populations. NATURALIS exhibited comparable efficacy shortly after application. Petir Kilat also demonstrated strong effectiveness potential, especially at higher concentrations. RLs displayed dose-dependent activity. They increased at higher concentrations and with repeated applications. While not as potent as traditional pesticides, RLs show promise as a component of integrated pest management strategies, particularly when combined with other control measures. The study also underscores the potential of essential oil-based products like Petir Kilat as sustainable alternatives in pest control.

**Abstract:**

Rhamnolipids (RLs), biosurfactants produced by *Pseudomonas aeruginosa*, have gained attention for their potential role in pest management. This study investigated the efficacy of RLs in controlling the two-spotted spider mite (*Tetranychus urticae*) and the whitefly (*Trialeurodes vaporariorum*), as well as a novel non-commercial essential oil-based product, Petir Kilat, on cucumber and tomato plants within a controlled greenhouse environment. The RLs were tested at concentrations of 1 mL/L and 2 mL/L, compared to commercial biopesticides including abamectin (ABAMAX) and Beauveria bassiana (NATURALIS). The results indicated that ABAMAX achieved the highest mortality rates for *T. urticae* and *T. vaporariorum*, with 100% mortality observed at 7 days. NATURALIS was also highly effective, particularly for whiteflies, though its efficacy declined over time. RLs showed a dose-dependent increase in mortality, with the higher concentration (2 mL/L) yielding more promising results, though not surpassing the commercial products. Petir Kilat, derived from orange essential oils, demonstrated significant control, particularly at higher concentrations, comparable to or exceeding the effectiveness of NATURALIS and ABAMAX in some cases. Statistical analyses revealed significant differences between treatments in most cases (*p* < 0.05). The findings underscore the potential of RLs and Petir Kilat as components of integrated pest management (IPM) strategies. While RLs are effective, their performance suggests they are best used in combination with other control methods. The study highlights the need for further research to optimize the application of RLs and essential oil-based products to enhance their role in sustainable pest management practices.

## 1. Introduction

The intensification of global agriculture has heightened the need for pest management strategies that effectively balance efficacy with environmental sustainability. With growing concerns about the long-term impacts of synthetic pesticides on ecosystems and human health, there has been a shift toward exploring alternative approaches [1]. This includes the development and use of bio-rational pesticides, which promise reduced environmental side effects and slower resistance development among target pest populations [2].

Rhamnolipids (RHLs), a class of biosurfactants produced by the bacterium *Pseudomonas aeruginosa* and some *Burkholderia* species, have emerged as a promising tool in this context [3]. Known for their ability to disrupt cellular membranes, rhamnolipids alter membrane permeability by integrating into lipid bilayers, leading to cell lysis. This disruption enhances the effectiveness of other antimicrobial agents and can activate defensive plant responses by triggering key signaling pathways [4]. Due to these properties, rhamnolipids are considered potent against a variety of microbial pathogens and have potential applications in pest management [5,6].

Initially, rhamnolipids were applied primarily for their antifungal properties, demonstrating effectiveness against plant pathogens such as *Botrytis cinerea* in crops like grapes, as well as *Pythium capsici*, *Pythium aphanidermatum*, and *Plasmopora* sp. [7]. Their success in controlling fungal diseases established them as potential biocontrol agents beyond just fungicides. Moreover, rhamnolipids have shown promise in biomedical applications such as antibacterial, antifungal, antiviral, antiadhesive, and antiproliferative agents [8].

The current research has extended the use of rhamnolipids to controlling agricultural pests, including the two-spotted spider mite (*Tetranychus urticae*) and the whitefly (*Trialeurodes vaporariorum*), which are significant threats to crop productivity. *Tetranychus urticae* (two-spotted spider mite) and *Trialeurodes vaporariorum* (greenhouse whitefly) are major pests in greenhouse cucumbers and tomatoes, causing significant economic damage. *T. urticae* feeds on plant tissues, leading to chlorosis and reduced yields, while *T. vaporariorum* damages plants by sap-feeding and transmitting plant viruses. Both pests are challenging to control due to their rapid reproduction and resistance to chemical pesticides. The sustainable management of these pests is crucial for minimizing economic losses and reducing pesticide resistance. Evaluating biopesticides like rhamnolipids and Petir Kilat offers an eco-friendly solution, providing effective pest control while preserving greenhouse ecosystem health.

While some studies highlight the efficacy and safety of rhamnolipids [5], others call for further research to understand their mechanisms and optimize their use in various agricultural settings [9]. Yet, under these settings, the comparative effectiveness of rhamnolipids versus established bio-rational pesticides, such as abamectin and *Beauveria bassiana* (NATURALIS SC), as well as novel non-commercial compounds like Petir Kilat, remains under investigation.

Moreover, Petir Kilat, a novel non-commercial insecticidal product derived from orange essential oil, demonstrates promising insecticidal properties due to its rich terpenoid content. Terpenoids, as secondary plant metabolites, contribute to their insecticidal, antimicrobial, and antioxidant effects [10,11]. Petir Kilat targets specific pest life stages and disrupts their physiological functions, positioning it as a potential environmentally friendly alternative to traditional pesticides. However, its mode of action, compatibility with beneficial insects, and potential non-target impacts require comprehensive research. This study aims to evaluate the efficacy of rhamnolipids and Petir Kilat against *T. urticae* and *T. vaporariorum* on greenhouse cucumbers and tomatoes. It also compares their performance with conventional bio-rational pesticides, such as abamectin and *Beauveria bassiana*, and the novel non-commercial essential oil-based product Petir Kilat. The research will contribute to the ongoing debate on the viability of rhamnolipids as biocontrol agents and their role within integrated pest management (IPM) frameworks. By providing a detailed comparative analysis, this study seeks to offer insights into the dose-dependent effectiveness of rhamnolipids and Petir Kilat, supporting informed decision making in sustainable agricultural practices and crop protection.

## 2. Materials and Methods

### 2.1. Plant Material and Pest Identification

Tomato (*Solanum lycopersicum*) and cucumber (*Cucumis sativus*) seedlings of similar size and developmental stage were obtained from a local nursery, transplanted into pots with a standardized potting mix, and maintained in a controlled greenhouse environment with a set temperature (26 °C), humidity (65%), and photoperiod (16:8 h) until the experiment began.

Identification of *Tetranychus urticae* was based on three morphological characteristics: (i) body color, with *T. urticae* typically appearing yellow–brown or greenish-brown and displaying two dark spots on the abdomen, while *Tetranychus evansi*, which might also attack tomatoes, is generally orange or reddish-orange [12], and (ii) the empodium. In *Tetranychus*, the lateral claws are reduced to small pads, each with a pair of long tenent hairs, whereas *T. evansi* has a typical claw-like empodium I and II [13] and (iii) the shape of the male’s aedagus, which differs between the two species, with *T. urticae* characterized by a large posterior projection [12,13]. Representative samples were brought to the laboratory for microscopic examination of male specimens to confirm species identification.

*Trialeurodes vaporariorum* was identified based on its dorsal surface, color, size, and wing shape [14]. These morphological characteristics can be used for adult identification in field conditions to distinguish it from *Bemisia tabaci*. *T. vaporariorum* is generally larger than *Bemisia tabaci* and has a triangular wing posture at rest, while *B. tabaci* has a more acute linear wing posture [15]. The puparium stage of *T. vaporariorum* was also used for identification, as it can be easily distinguished from *B. tabaci* by its palisade of wax that gives it a ‘pill-box’ appearance. Additionally, the outline of *T. vaporariorum* is not distorted by plant hairs, as is often seen in *B. tabaci*, and there is an absence of dorsal setae when it is viewed under a ×20 hand lens [16].

### 2.2. Standardization of the Spraining Protocol

The spraying duration was standardized using a backpack automated air-assisted electrostatic spraying apparatus (Chemex Industry, Inc., Irvine, CA, USA), where each plant was sprayed individually until water droplets began to form and leak from the leaves. During operation, the air moved through the nozzle at high speed and intersected with the liquid at the nozzle tip, causing the formation of spray droplets 30 to 60 microns in diameter. The air pressure required for all treatments ranged from 15 to 60 psi, and the liquid pressure was set to below 30 psi. This ensured consistent coverage across all treated plants and repeatability of treatments, as the force of the turbulent air stream propelled the charged droplets deep into the plant canopy in the same manner for each treatment.

Before the bioassays, the spraying apparatus was calibrated with water to standardize droplet size and pressure. This ensured that the spraying conditions did not impact pest mortality, as confirmed by the absence of effects on pests exposed to water alone with 0.03% Tween 80^®^. Furthermore, no phytotoxic effects were observed during preliminary field tests, validating the safety of the treatments and eliminating the need for a water-only control.

### 2.3. Biorational Test Compounds

- Rhamnolipids (RHLs)

**Figure 1:**
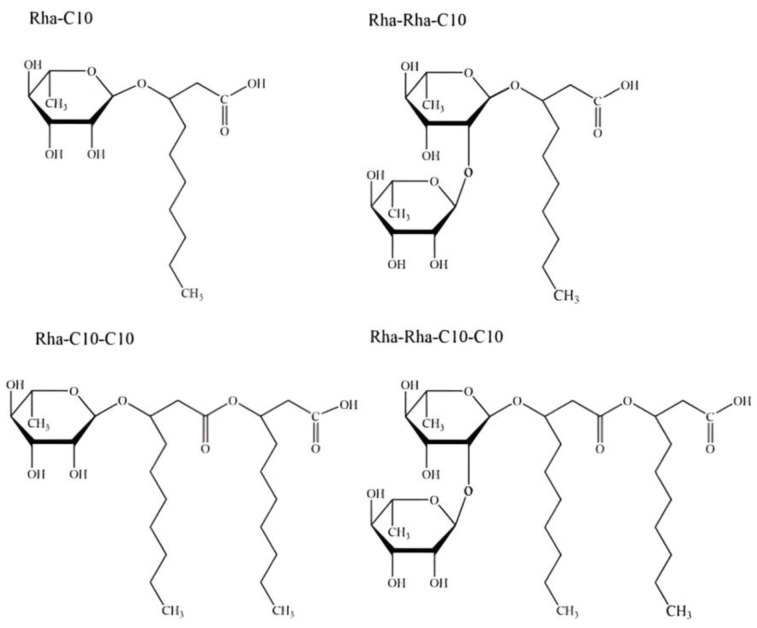
Common structure of different rhamnolipids types (RL-1 to RL-4) produced by *Pseudomnas* spp. [18] (Available via license: CC BY 4.0). Name classification according to IUPAC of the first identified rhamnolipid (R1) named as 3-[3-[(2R,3R,4R,5R,6S)-4,5-dihydroxy-6-methyl-3-[(2S,3R,4R,5R,6S)-3,4,5-trihydroxy-6-methyloxan-2-yl]oxyoxan-2-yl]oxydecanoyloxy]decanoic acid (computed by Lexichem TK 2.7.0—PubChem release 14 October 2021) [19].

Commercially available RHL solutions were obtained from Printzstraße 4, 761,39 Karlsruhe, Germany. The product specifications indicated a mixture of mono- and di-rhamnolipid congeners, simply named α-L-rhamnopyranosyl-α-L-rhamnopyranosyl-β-hydroxydecanoyl-β-hydroxydecanoate and symbolized as Rha-Rha-C10-C10 with an average molecular mass of RHLs ranges between 302 and 989 Da. The RHL solutions were prepared at concentrations of 1 mL/L and 2 mL/L using sterile distilled water. Fresh solutions were prepared for each application to minimize degradation. The chemical structure of the first identified rhamnolipid is shown in Figure 1.

- Macrolitic Lactones (MLs)

**Figure 2:**
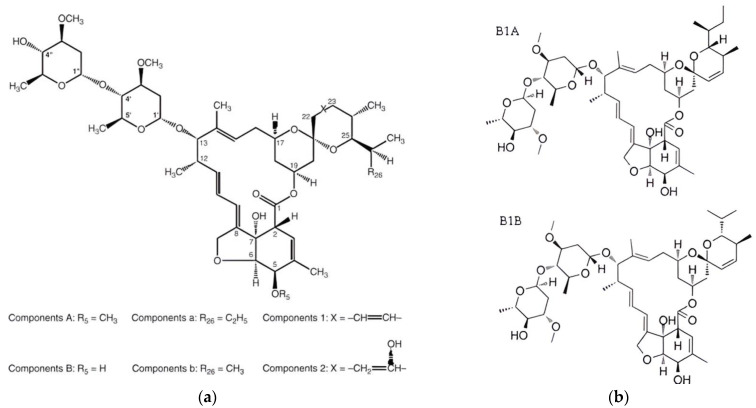
(**a**) Chemical structure of the general avermectin class [20] (**a**,**b**) chemical structure of Avermectin B1 or abamectin (80% of the B1A homolog and not more than 20% of the B1B homolog) [21]. The name classification of B1A is (1′R,2R,3S,4′S,6S,8′R,10′E,12′S,13′S,14′E,16′E,20′R,21′R,24′S)-2-[(2S)-butan-2-yl]-21′,24′-dihydroxy-12′-[(2R,4S,5S,6S)-5-[(2S,4S,5S,6S)-5-hydroxy-4-methoxy-6-methyloxan-2-yl]oxy-4-methoxy-6-methyloxan-2-yl]oxy-3,11′,13′,22′-tetramethylspiro [2,3-dihydropyran-6,6′-3,7,19-trioxatetracyclo [15.6.1.1^4,8^.0^20,24^]pentacosa-10,14,16,22-tetraene]-2′-one (computed by Lexichem TK 2.7.0—PubChem release 14 October 2021).

Abamectin, a commercially available miticide belonging to the avermectin class, was used as a reference treatment. The product, ABAMAX 2.5SC [Abamectin (aka avermectin 2.61% *w*/*o*)], was obtained from ADAMA Hellas, Monumental Plaza, Leoforos Kifisias adrs. 44, Marousi Athens 151,25, Greece. A spray solution was prepared according to the manufacturer’s instructions for application to target pests. The chemical structure of avermectins, which belong to the chemical class of macrocyclic lactones, a group of naturally occurring antibiotics produced by soil actinomycete *Streptomyces avermitilis* [17], is shown in Figure 2. The use of Avermectin B1 or abamectin (80% of the B1A homolog and not more than 20% of the B1B homolog).

- Petir Kilat (PK)

**Figure 3:**
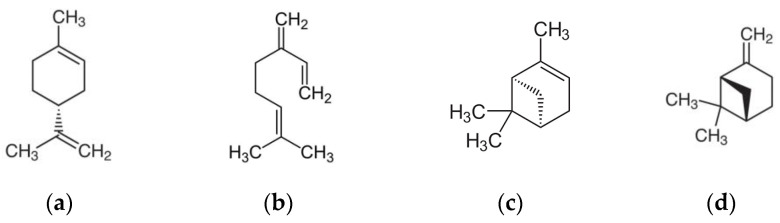
Chemical structure of the major essential oils terpenoid contents of Petir Kilat and related IUPAC names (**a**) d-limonen [(R)-4-isopropenyl-1-methylcyclohexene or p-mentha-1,8-diene], (**b**) myrcene [7-methyl-3-methylideneocta-1,6-diene], (**c**) a-pinene [(1S,5S)-2,6,6-Trimethylbicyclo [3.1.1]hept-2-ene], and (**d**) b-pinene [6,6-dimethyl-2-methylidenebicyclo [3.1.1]heptane] (computed by Lexichem TK 2.7.0—PubChem release 14 October 2021) [19].

Petir Kilat is a novel non-commercial compound derived from orange essential oils (EOs). Orange essential oil is a complex mixture of terpenoids, primarily composed of monoterpenes and sesquiterpenes, which exhibit various biological activities, including insecticidal and acaricidal properties. D-limonene, the most abundant and active monoterpene, disrupts insect and mite physiology through neurotoxicity. Other terpenes, such as myrcene and b-pinene, also contribute to insecticidal effects by interfering with respiration, nerve function, and hormone regulation (Figure 3).

- Beauveria bassiana—BioCide

A commercially available bioinsecticide, NATURALIS SC, based on the entomopathogenic fungus *Beauveria bassiana* strain ATCC 74040, was used as a second reference treatment for comparing the efficacy of rhamnolipids and Petir Kilat (PK). The product was obtained from INTRACHEM HELLAS, Kifisias 31 adrs., Athens, 115,23, Greece, and applied according to the manufacturer’s recommendations.

### 2.4. Bioassays, Experimental Set Up, and Data Collection

Bioassays were performed on two pests, *T. urticae* and *T. vaporariorum*, which are indigenous and have consistently been present in the greenhouse and classified over the previous years. Additionally, the greenhouse has not undergone any prior treatments with synthetic pesticides, as it is managed as a biological greenhouse. This natural presence eliminated the need for the artificial introduction of pests, thereby ensuring the authenticity of the study conditions. Moreover, although we did not standardize the life stages for the initial infestation, we carefully monitored and examined multiple developmental stages throughout the study. Our focus was on all mobile stages of *T. urticae* and *T. vaporariorum*. Specifically, for *T. vaporariorum*, this included both larvae (crawlers) and adults, allowing us to comprehensively assess the effects of the treatments across these critical life stages. Host samples were monitored regularly after biocide applications. Mortality for *Trialeurodes vaporariorum* adults, some of which may have escaped during spraying, was assessed based on the individuals remaining on the host plants. In all cases, mortality was assessed by visually counting dead individuals under a stereoscope, using a fine-tipped brush (size 000) to gently handle any moving specimens and determine their status. We initially selected specific concentrations of RHs and Petir Kilat EOs based on preliminary trials within the typical range for similar biocides based on established guidelines or recommendations. These trials helped us determine effective and practical application rates for testing the compounds.

#### 2.4.1. Efficacy of RHs against *T. uritcae* and *T. vaporariorum*

The experiment utilized a completely randomized design with three replicates per treatment, each containing three tomato and cucumber plants. Five treatments were applied separately to the tomato and cucumber plants: (a) untreated controls, (b) plants treated with RHLs at 1 mL/L, (c) plants treated with RHLs at 2 mL/L, (d) plants treated with NATURALIS, and (e) plants treated with Abamectin 1.8 EC (reference product) at the recommended dose. Cucumber plants received the same treatments except for Naturalis and additionally underwent two spray applications one week apart. Mortality data were collected by recording the percentage of dead mites five days after each spray application on both tomato and cucumber plants. The mortality rates were assessed five and seven days after application for *T. urticae* and five days for *T. vaporariorum*. Only mobile stages were considered: nymphs and adults for *T. urticae* and larvae (crawlers) and adults for *T. vaporariorum*. This involved visually inspecting a predetermined number of leaves per plant and counting the number of dead mites/whiteflies compared to the initial population size using a stereoscope.

#### 2.4.2. Efficacy of Petir Kilat against *T. urticae* and *T. vaporariorum*

In a separate set of experiments, we tested the comparative efficacy of Petir Kilat and ABAMAX as baselines against *T. urticae*, as well as Petir Kilat and NATURALIS as baselines against *T. vaporariorum* on cucumber plants. We applied the same experimental randomized design with three replicates for each treatment as described in 2.2.1. There were four treatments: (a) untreated control plants, (b) plants treated with Petir Kilat at a rate of 1%, (c) plants treated with Petir Kilat at a rate of 1.2%, and (d) plants treated with ABAMAX (reference product) at the recommended dose in the bioassays with *T. urticae* and with NATURALIS SC (reference product) in the bioassays with *T. vaporariorum*. Two successive spray applications were conducted, with mortality assessments performed five days after each application. The percentage mortality was calculated by counting the number of dead whiteflies compared to the initial population size using a stereoscope.

### 2.5. Statistical Analysis

Statistical analysis involved assessing the normality of mortality data for each treatment, plant type (tomato and cucumber), and the two-time points for each case using visual inspection and the Shapiro–Wilk test (α = 0.05) [22]. Arcsine Square Root Transformation was performed if data did not meet normality assumptions and a one-way ANOVA was conducted for each plant type and time point to compare treatment means. Significant differences (*p* < 0.05) identified by the ANOVA were further analyzed using Duncan’s Multiple Range Test (α = 0.05) (Tallarida and Murray, 1987) [23]. This test employs a critical difference (CD) calculated as the product of a critical value (q) obtained from the studentized range distribution table (based on degrees of freedom within groups) and the square root of the mean squares within groups (MSW) from the ANOVA, divided by the number of observations per group (replicates). Treatment means with a difference exceeding the calculated CD were considered statistically different in mortality rates. To manage false positives, we used an FDR correction instead of the more conservative Bonferroni test, which can be overly conservative when the number of comparisons is large.

## 3. Results

### 3.1. Comparative Efficacy of RHLs and Commercial Biocides against T. urticae and T. vaporariorum of Tomato Plants

**Figure 4:**
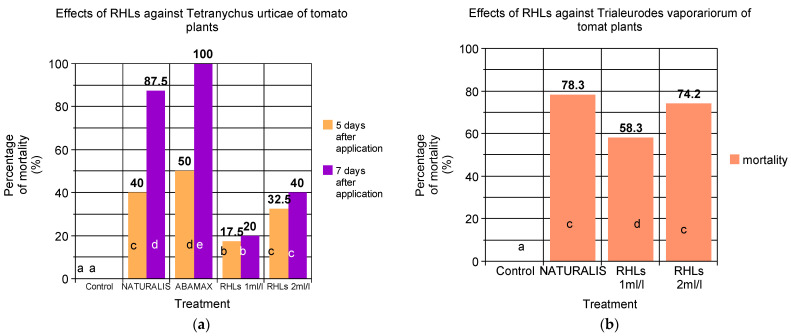
(**a**) Comparative efficacy of two different doses (1 mL/L and 2 mL/L) of RHLs and two commercial biorational products, NATURALIS and ABAMAX, against *T. urticae* on tomato plants recorded 5 and 7 days after spray application, respectively. (**b**) Comparative efficacy of two different doses (1 mL/L and 2 mL/L) of RHLs and the commercial product NATURALIS against the whitefly *T. vaporariorum* on tomato plants. Plants did not receive any spray application until the first population of *T. urticae* was observed. Values in bars with the same color (one-way ANOVA) followed by the same letter are not significantly different (*p* < 0.05) according to Duncan’s Multiple Range Test.

The results of the study on the effects of RHLs against *T. urticae* on tomato plants are shown in Figure 4a. As anticipated, tomato plants of the control group showed no *T. urticae* mortality across both 5-day and 7-day intervals. The commercial product NATURALIS resulted in a significant increase in mortality, with 40% mortality at 4 days and 87.5% at 7 days. ABAMAX, another commercial product, achieved the highest mortality rates, with 100% at 7 days, yet no effectiveness was observed at 4 days after spray application. The RHL treatments displayed a dose-dependent effect. The RHLs at 1 mL/L concentration demonstrated 17.5% mortality at 4 days and 20% at 7 days after application. Doubling the concentration to 2 mL/L improved effectiveness, leading to 32.5% mortality at 4 days and 40% at 7 days after spray application. Overall, the insecticide Abamectin (ABAMAX) proved to be the most effective treatment against *T. urticae*. While Naturalis offered comparable effectiveness to Abamectin at the 4-day evaluation, its effectiveness waned at 7 days. Similarly, the RHLs at 2 mL/L concentration achieved similar mortality rates to Naturalis at 4 days, but their effectiveness also declined at 7 days. Overall, both RHL concentrations increased mortality compared to the control group, but they consistently fell short of the efficacy achieved by the commercial products.

The insecticidal efficacy of various treatments against the greenhouse whitefly, *T. vaporariorum*, on tomato plants was evaluated and is shown in Figure 4b. Mortality rates were assessed and statistically analyzed using Duncan’s Multiple Range Test (α = 0.05). The commercial product NATURALIS demonstrated the highest level of control, achieving a significant mortality rate of 78.3%. RHLs displayed a dose-dependent effect. The lower concentration (1 mL/L) resulted in a moderate but significant increase in mortality, reaching 58.3%. Doubling the concentration to 2 mL/L led to improved effectiveness, with a significant mortality rate of 74.2%, comparable to NATURALIS. Duncan’s Multiple Range Test revealed that NATURALIS and RHLs at 2 mL/L were statistically the most effective treatments, significantly outperforming the untreated control and RHLs at 1 mL/L. While the lower RHL concentration caused a significant increase in mortality compared to the control, its efficacy was lower than both NATURALIS and the higher RHL concentration. Overall, NATURALIS and RHLs at 2 mL/L emerged as the most effective treatments against *T. vaporariorum* on tomato plants without significant differences between them. This highlights the potential of RHLs as a control method, with their effectiveness increasing at higher concentrations.

### 3.2. Comparative Efficacy of RHLs and Commercial Biocide against T. urticae on Cucumber Plants

**Figure 5:**
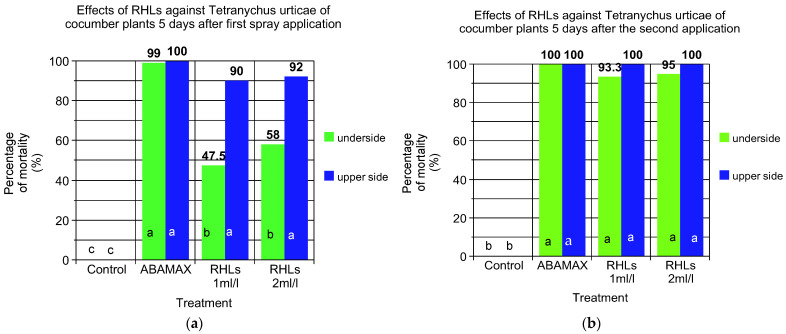
Comparative efficacy of two different doses (1 mL/L and 2 mL/L) of RHLs and Abamectin (commercial product ABAMAX) against *T. urticae* on cucumber plants recorded on the underside and upper side of the leaves; (**a**) 5 days after the first spray application and (**b**) 5 days after the second spray application. Plants did not receive any spray application until the first population of *T. urticae* was observed. Values in the same bar followed by the same letter are not significantly different (*p* < 0.05) according to Duncan’s Multiple Range Test.

Figure 5a,b depicts the effects at two RHL concentrations (1 mL/L and 2 mL/L) and the commercial product ABAMAX (Abamectin 1.8 EC) against *T. urticae* on cucumber plants recorded on the underside and the upper side of the leaves, respectively. The control group exhibited no mortality at both the 4-day and 7-day evaluations, confirming the absence of natural mortality or other influencing factors (Figure 5). Abamectin demonstrated the highest efficacy in controlling *T. urticae*, achieving a 100% mortality rate at the 7-day evaluation. However, no mortality was observed at the 4-day mark, indicating that its effects became pronounced only after a longer exposure period. The RHL treatments showed promising potential in controlling *T. urticae*. The 1 mL/L concentration resulted in a moderate increase in mortality rates, with 17.5% at the 4-day evaluation and 20% at the 7-day mark. Doubling the concentration of RHL enhanced its effectiveness significantly. At 4 days, the mortality rate was 32.5%, which further increased to 40% at the 7-day evaluation. Additionally, there were no significant differences in the recorded mortality rates on the undersides (Figure 5a) versus the upper sides (Figure 5b) of the cucumber leaves, indicating uniform effectiveness of Abamectin on different leaf surfaces but not for RHLs. Overall, while Abamectin (Abamectin 1.8 EC) was the most effective treatment against *T. urticae*, achieving complete control by the end of the 7-day period, the RHL treatments demonstrated a dose-dependent response and considerable potential. The higher concentration (2 mL/L) provided better control than the lower concentration (1 mL/L), indicating that with further optimization, RHL biocides could be a viable alternative for managing *T. urticae* infestations.

### 3.3. Comparative Efficacy of PK and Commercial Biocides against T. urticae on Cucumber Plants

**Figure 6:**
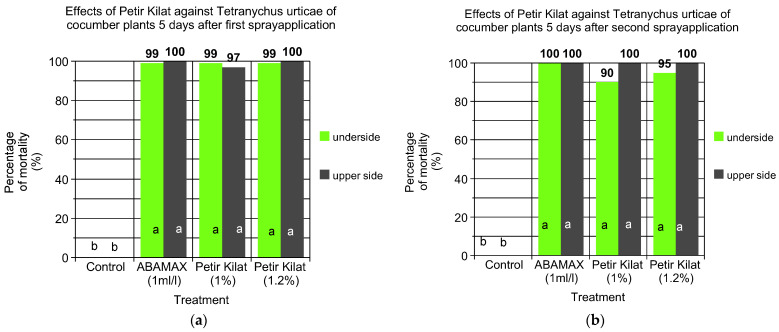
Comparative efficacy of two different doses (1% and 1.2% *w*/*o*) of Petir Kilat and Abamectin (commercial product ABAMAX) against *T. urticae* on cucumber plants recorded on the underside and upper side of the leaves; (**a**) 5 days after the first spray application and (**b**) 5 days after the second spray application. Plants did not receive any spray application until the first population of *T. urticae* was observed. Values in the same bar followed by the same letter are not significantly different (*p* < 0.05) according to Duncan’s Multiple Range Test.

The effects of Petir Kilat and ABAMAX against *T. urticae* on cucumber plants were evaluated 5 days after the first spray application and the results are shown in Figure 6a. The treatments included ABAMAX at 1 mL/L, Petir Kilat at 1%, Petir Kilat at 1.2%, and an unsprayed control. ABAMAX at a concentration of 1 mL/L achieved a mortality rate of 99% on the underside of the leaves and 100% on the upper side. Similarly, Petir Kilat, at both concentrations tested (1% and 1.2%), resulted in high mortality rates. At 1%, Petir Kilat showed 99% mortality on the underside and 97% on the upper side of the leaves. When the concentration was increased to 1.2%, mortality rates were 99% on the underside and 100% on the upper side of the leaves. In contrast, the unsprayed control group exhibited no mortality on either the underside or upper side of the leaves. Statistical analysis using Duncan’s Multiple Range Test (*p* < 0.05) indicated that there were no significant differences in mortality rates between the ABAMAX and Petir Kilat treatments at both concentrations, while all treatments significantly outperformed the unsprayed control. This demonstrates that both ABAMAX and Petir Kilat are highly effective in controlling *T. urticae* in cucumber plants.

Figure 6b shows the effects of Petir Kilat and ABAMAX against *T. urticae* on cucumber plants that were evaluated 5 days after the second spray application. ABAMAX, at a concentration of 1 mL/L, achieved 100% mortality on both the underside and upper side of the leaves. Petir Kilat, at 1%, resulted in 90% mortality on the underside and 100% on the upper side of the leaves. Increasing the concentration of Petir Kilat to 1.2% improved the mortality rate to 95% on the underside while maintaining 100% on the upper side of the leaves. The unsprayed control group exhibited no mortality on either the underside or upper side of the leaves. There were no significant differences in mortality rates among the ABAMAX and Petir Kilat treatments at both concentrations, while all treatments significantly outperformed the unsprayed control. These results demonstrate the high effectiveness of Petir Kilat, similar to the commercial compound ABAMAX in controlling *T. urticae* on cucumber plants, particularly on the upper side of the leaves where both achieved 100% mortality.

### 3.4. Comparative Efficacy of PK and Commercial Biocides against T. vaporariorum on Cucumber Plants

**Figure 7:**
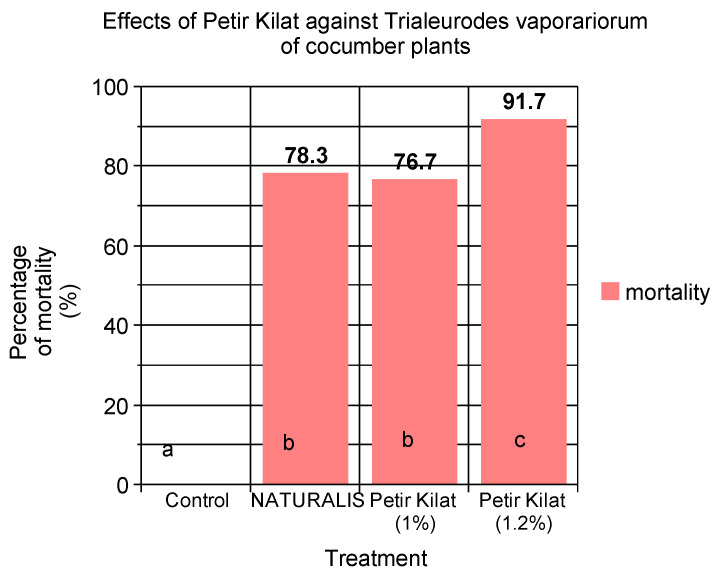
Comparative efficacy of two different doses (1% and 1.2% *w*/*o*) of Petir Kilat and NATURALIS against *T. vaporariorum* on cucumber plants recorded 5 days after the first spray application. Plants did not receive any spray application until the first population of *T. urticae* was observed. Values in the same bar followed by the same letter are not significantly different (*p* < 0.05) according to Duncan’s Multiple Range Test.

The comparative insecticidal efficacy of Petir Kilat and NATURALIS against *T. vaporariorum* was also evaluated, and the results are presented in Figure 7. Both insecticides, when applied at a concentration of 1%, exhibited significantly higher control efficacy compared to the untreated control, indicating their effectiveness in managing *T. vaporariorum* populations. However, Petir Kilat, when applied at a concentration of 1.2%, demonstrated an even higher level of control, significantly surpassing both the 1% treatments and the untreated control. This suggests that increasing the concentration of Petir Kilat enhances its insecticidal activity. No significant difference in efficacy was observed between NATURALIS and Petir Kilat when both were applied at the 1% rate, indicating that both products are equally effective at this concentration. These findings highlight the potential of Petir Kilat as a potent insecticide, particularly at higher concentrations, and underscore the importance of optimizing dosage for effective pest management.

## 4. Discussion

The exploration of rhamnolipids (RHLs) as biocontrol agents in pest management has revealed promising outcomes, albeit with limitations compared to conventional insecticides. Rhamnolipids, primarily known as biosurfactants produced by bacteria such as *Pseudomonas aeruginosa*, have shown efficacy in controlling pest populations through mechanisms distinct from traditional insecticides. Research indicates that RHLs can act as growth inhibitors and repellents and can even induce mortality [24,25], as shown in the current study, in certain pest species, such as the two-spotted spider mite (*Tetranychus urticae*) and the greenhouse whitefly (*Trialeurodes vaporariorum*).

In our study, RHLs demonstrated a dose-dependent insecticidal effect against *T. urticae* on tomato plants. The RHL treatments at 1 mL/L and 2 mL/L resulted in 17.5% and 32.5% mortality at 4 days, respectively, increasing to 20% and 40% at 7 days. These results suggest that higher concentrations of RHLs enhance their effectiveness, although they consistently fell short of the efficacy achieved by commercial products such as NATURALIS and ABAMAX. NATURALIS and ABAMAX displayed significantly higher mortality rates, with NATURALIS achieving 87.5% mortality at 7 days and ABAMAX achieving 100% mortality by the same interval. Similarly, RHLs showed effectiveness against *T. vaporariorum* on tomato plants. At a concentration of 1 mL/L, RHLs achieved a 58.3% mortality rate, which increased to 74.2% at 2 mL/L. This dose-dependent response indicates the potential of RHLs as a biocontrol agent. However, NATURALIS, with a mortality rate of 78.3%, outperformed RHLs at the 1 mL/L concentration but showed comparable efficacy at the higher RHL concentration.

Our findings are consistent with previous studies demonstrating the effectiveness of essential oils in controlling related pest species, such as *Bemisia tabaci*, highlighting their potential as valuable alternatives to chemical control and their role in integrated pest management strategies [26].

The results presented for Petir Kilat align with the growing body of research highlighting the potential of EOs and their constituent terpenoids as effective insecticides (to mention only a few see Regnault-Roger et al. 2012, Dambolena 2016, and Liu et al., 2023) [27,28,29]. These natural compounds have demonstrated insecticidal properties through various mechanisms, including disruption of the insect nervous system [30], interference with hormone regulation acting as Insect Growth Regulators [10], and repellent effects [31,32].

The evaluation of Petir Kilat, a novel non-commercial insecticidal product based on the essential oils of orange, revealed highly promising results. Petir Kilat’s performance was comparable to that of the commercial insecticides ABAMAX and NATURALIS in controlling *T. urticae* and *T. vaporariorum*. Against *T. urticae* on cucumber plants, Petir Kilat, at both 1% and 1.2%, concentrations demonstrated high mortality rates, comparable to ABAMAX, which achieved near-complete control. This high level of efficacy was maintained across both the upper and underside of leaves, highlighting the uniform effectiveness of Petir Kilat. Against *T. vaporariorum*, Petir Kilat exhibited significant control, with increased efficacy at a higher concentration (1.2%). The lack of significant differences between NATURALIS and Petir Kilat at the 1% rate underscores the potential of Petir Kilat as an effective biocontrol agent.

Additionally, essential oils (EOs) have shown synergistic effects that can reduce the risk of resistance development, making them promising ingredients for new biopesticides. However, while EOs are generally considered safer for non-target organisms, some can negatively impact beneficial control agents, such as predatory insects and parasites, affecting their respiration, predatory ability, and parasitization rates, among other factors [33]. Therefore, it is crucial to consider the potential impacts of essential oils, including those in Petir Kilat, on beneficial insects and other non-target organisms. These effects may include sub-lethal impacts, such as reduced reproduction rates and impaired development, which could disrupt ecosystem balance and harm beneficial insect populations [34]. EOs can have variable impacts on pollinators, which are vital for ecosystem health and agriculture. While some EOs may act as attractants or have minimal effects, others can be detrimental affecting crop yields [35]. Additionally, challenges such as limited biomass availability, chemical stability, formulation difficulties, and phytotoxicity remain obstacles to the widespread adoption of EO-based biopesticides [36].

This highlights the importance of optimizing dosage for maximizing pest control efficacy. Thus, the comparable efficacy of Petir Kilat to synthetic insecticides like ABAMAX is particularly noteworthy. In addition, the doses that have been used are in accordance with similar works that have tested the insecticidal properties of essential oil vapors from *Satureja hortensis* L., *Ocimum basilicum* L., and *Thymus vulgaris* L. (Lamiacae) were tested for their toxicities against the nymphs and adults of *Tetranychus urticae* Koch (Acari: Tetranychidae) and adults of *Bemisia tabaci* Genn. (Homoptera: Aleyrodidae) [37].

Similarly, in the search for environmentally friendly control measures against the tomato red spider mite (*Tetranychus evansi*) under greenhouse conditions, Organic Neem and Natuneem also demonstrated promising results, although the latter caused phytotoxicity. This highlights the importance of continued research into viable alternatives to synthetic compounds for pest control. Research on alternative strategies for controlling *T. vaporariorum* emphasizes the potential of exploring novel biological agents such as rhamnolipids and essential oil-based products [38]. Moreover, with regard to other biorational compounds, not all strains of the entomopathogenic fungus *Beauveria bassiana* are equally effective against pests including the whitefly *Bemisia tabaci* and other species, highlighting the importance of careful strain selection for optimal pest control [39].

These findings reinforce the necessity of ongoing research into innovative sustainable solutions for pest control, suggesting that the Petir Kilat essential oil-based product can provide a viable alternative to conventional pesticides, offering potential benefits such as reduced environmental impact and lower toxicity to non-target organisms. The broad-spectrum activity of Petir Kilat, as demonstrated by its effectiveness against both *T. urticae* and *T. vaporariorum*, further emphasizes its potential as a versatile pest management tool.

To put forward the uniform efficacy of Petir Kilat on both leaf surfaces is indicative of its good coverage and penetration, crucial factors for effective pest control. This characteristic, combined with its high mortality rates, suggests that Petir Kilat has the potential to be a highly efficient insecticide for managing populations of *T. urticae* and potentially other pest species.

While RHLs and Petir Kilat show potential, their role is best suited within an integrated pest management (IPM) strategy rather than as standalone insecticides. The effectiveness of RHLs can be influenced by concentration and frequency of application, and they often perform better when combined with other control methods. This multifaceted approach can reduce the reliance on traditional chemical pesticides, contributing to more sustainable agricultural practices [40].

The utilization of biosurfactants such as rhamnolipids and essential oil-based formulations like Petir Kilat signifies a paradigm shift toward more environmentally benign pest management strategies. As demonstrated in this study, these biological agents offer promising alternatives to synthetic pesticides, with the potential to mitigate environmental impact and promote sustainable pest control practices. Thus, the potential compatibility of RHLs and essential oil-based products with greenhouse pest management systems is promising. Their incorporation into IPM strategies can be particularly beneficial in greenhouse environments, where maintaining a balance between pest control and the preservation of beneficial organisms is crucial. Moreover, these biocontrol agents can be integrated with biological control methods to enhance overall pest management efficacy and sustainability.

On the other hand, future research should focus on optimizing the application methods and concentrations of RHLs and essential oils to maximize their efficacy. Studies could also explore the synergistic effects of combining these biocontrol agents with other IPM components. Additionally, understanding the long-term impacts of these biocontrol agents on non-target organisms and the environment will be essential for their successful integration into sustainable pest management practices. For instance, even at non-lethal concentrations, RHLs and EOs can induce sub-lethal effects, such as reduced reproduction rates, impaired development, and weakened immune systems in non-target arthropods, including natural pollinators. However, there is limited specific information on their effects on beneficial species in tomato and cucumber greenhouse environments. To mitigate these risks, RHLs and EOs can be applied in a targeted manner to minimize exposure to non-target species, such as using precision spraying techniques and restricting applications to pest-damaged plants or times when non-target species are less active. Additionally, EOs offer an environmentally friendly alternative to more persistent synthetic pesticides, reducing the risk of chemical residue accumulation in the ecosystem [41].

Finally, the controlled greenhouse environment in this study likely contributed to both the observed variability and the high efficacy of rhamnolipids (RLs) and Petir Kilat. The stable microclimate, with minimal external stressors, allowed for a clearer evaluation of the agents’ effectiveness. For instance, ABAMAX achieved 100% mortality against *T. urticae* after 7 days, while RL treatments showed a dose-dependent response, reaching 40% mortality at the higher concentration. Similarly, Petir Kilat (1% and 1.2%) resulted in near-complete mortality (99–100%) on both leaf surfaces, highlighting the favorable conditions provided by the greenhouse.

However, field conditions—with their greater variability in pest populations and environmental factors—may alter the effectiveness of RLs, Petir Kilat, and commercial biocides [42]. Future studies should replicate these experiments in field settings to assess the consistency and reliability of these biocontrol agents under real-world conditions. Moreover, the surrounding landscape of small greenhouse settings might have contrasting and specific effects on crop colonization by different pest and biological control agent groups, as well as on the initial pest status and their management [43]. Nonetheless, despite the small-scale nature of our greenhouse environment, which may limit the generalizability of our findings to larger more variable environments, the results provide promising evidence for the potential of RLs and Petir Kilat as alternatives to commercial biocides and conventional products for sustainable pest management. In conclusion, while commercial products like ABAMAX and NATURALIS remain highly effective against *T. urticae* and *T. vaporariorum*, the incorporation of rhamnolipids and Petir Kilat into pest management strategies shows considerable promise. Further research and optimization of these biocontrol agents could enhance their efficacy, making them valuable components of integrated pest management programs.

## Data Availability

Data are available upon request.
